# Impacts of Sol-Gel Auto-Combustion and Ultrasonication Approaches on Structural, Magnetic, and Optical Properties of Sm-Tm Co-Substituted Sr_0.5_Ba_0.5_Fe_12_O_19_ Nanohexaferrites: Comparative Study

**DOI:** 10.3390/nano10020272

**Published:** 2020-02-06

**Authors:** Yassine Slimani, Munirah Abdullah Almessiere, Sadik Güner, Umran Kurtan, Abdulhadi Baykal

**Affiliations:** 1 Department of Biophysics, Institute for Research and Medical Consultations (IRMC), Imam Abdulrahman Bin Faisal University, P.O. Box 1982, Dammam 31441, Saudi Arabia; malmessiere@iau.edu.sa; 2 Institute of Inorganic Chemistry, RWTH Aachen University, D-52074 Aachen, Germany; s.guner@ac.rwh-aachen.de; 3 Department of Materials and Materials Processing Technologies, Vocational School of Technical Sciences, İstanbul University-Cerrahpaşa, 34500 İstanbul, Turkey; umrankurtan@gmail.com; 4 Department of Nanomedicine, Institute for Research and Medical Consultations (IRMC), Imam Abdulrahman Bin Faisal University, P.O. Box 1982, Dammam 31441, Saudi Arabia; abaykal@iau.edu.sa

**Keywords:** hexaferrite, rare earths, structure, morphology, magnetic properties, optical properties

## Abstract

In this paper, we introduced a comparative study of Sm-Tm-substituted Sr-Ba nanohexaferrites (NHFs), Sr_0.5_Ba_0.5_Tm*_x_*Sm*_x_*Fe_12−2*x*_O_19_ with *x* = 0.00–0.05, manufactured via both citrate sol-gel auto-combustion and ultrasonication approaches. The phase formation of M-type hexaferrite (HF) for both compositions was confirmed by X-ray diffraction (XRD) powder pattern, Fourier-transform infrared (FT-IR) spectra, scanning and transmission electron microscopy (SEM and TEM) micrographs, energy dispersive X-ray (EDX) spectra, and elemental mappings. The magnetic properties at room temperature (RT) and low temperature (*T* = 10 K) were also investigated. M-H loops revealed ferrimagnetic nature for various prepared nanohexaferrites via sol-gel and ultrasonication routes. The *M_s_* (saturation magnetization) and *M_r_* (remanence) values increased with increasing Tm-Sm substituting contents. *M_s_* and *M_r_* reached their maximum values at *x* = 0.04 in the case of samples prepared using the sol-gel technique and at *x* = 0.03 for those prepared via ultrasonication route. *M-H* loops were very broad in samples prepared via ultrasonication route in comparison to those produced by means of the sol-gel approach, implying that the products synthesized via ultrasonication route have greater values of coercivity (*H_c_*). The variations in *H_c_* values with respect to Tm-Sm substitutions were governed by the evolutions in the magneto-crystalline anisotropy. Diffuse reflectance spectra (DRS) were employed to estimate the direct band gap of pristine and co-substituted Sr_0.5_Ba_0.5_Fe_12_O_19_ hexaferrites. Optical energy band gaps (*E*_g_) of pristine samples were significantly tuned by co-substitution of Tm^3+^ and Sm^3+^ ions. *E*_g_ values of the Sr_0.5_Ba_0.5_Fe_12_O_19_ sample, which was synthesized using the sol-gel method, decreased almost linearly from 1.75 to 1.45 eV by increasing co-doped ion content. However, we observed a sharp drop from 1.85 eV to an average of 1.50 eV for the samples, which were synthesized using the ultrasonication approach.

## 1. Introduction

Ferrite materials cover almost all the branches of material science. Among them, M-type barium (Ba) or strontium (Sr) ferrites (MFe_12_O_19_, where M = Ba, Sr, and Pb) with a hexagonal structure have been exclusively utilized in a wide range of technological applications since they possess unique magnetic and dielectric features [1,2,3]. They play a critical role in permanent magnets, data recording media, radar absorbing, sensors, telecommunications, and microwave devices because of their excellent chemical stability and low cost. Moreover, the large crystalline anisotropy, high saturation magnetization, and high coercivity make them excellent candidates with respect to other magnetic materials [4,5,6,7]. 

The substitution of cations at Fe^3+^ or Ba^2+^ sites is an efficient technique to alter the magnetic, physical, and electrical traits of hexaferrites (HFs) since magnetic and other features are correlated to the distribution of the doping ions on five crystallographic sites. Several studies have been done to substitute Fe^3+^ ions by various cations, for example, Al^+3^ [8], Cr^+3^ [9], Eu-Nd [10], Ga^+3^ [11], Bi-Cr [12], Zr-Zn [13], Ti-Ru [14], and Mo-Zn [15], and significant changes were seen in magnetic and electrical features of substituted hexaferrites. Additionally, the substitution of the rare earth (RE) elements such as Tb^+3^ [16] and Nb-Dy [17] has been investigated to enhance the electromagnetic response of hexaferrites. Z. Somogyvári et al. [18] studied the magnetic properties of Sc-substituted Ba-hexaferrites by using neutron diffraction and field-dependent ^57^Fe Mössbauer. From these measurements, the researchers showed how the added Sc atoms substitute for the five different Fe lattice sites. It was revealed that the RE (Sc in this case) atoms could substitute Fe lattice sites. Because the unpaired 4f electrons are present in the RE ions, the 4f–3d couplings of the angular momentum occur, resulting in electromagnetic property enhancement. Doping several elements into M-type hexaferrite can not only improve the magneto-crystalline anisotropy but also enhance the electrical and magnetic features in ferrite [19]. Hence, the characteristics of M-type hexaferrites can be changed by doping different cations into the structure [20]. 

Various studies have been done to improve the electromagnetic properties of ferrites by the replacement of Fe^3+^ with RE element cations. However, as far as we know, the simultaneous substitution of thulium-samarium (Tm-SM) into M-type Ba-Sr hexaferrites has not been reported until now. Accordingly, Ba_0.5_Sr_0.5_Tm*_x_*Sm*_x_*Fe_12−2*x*_O_19_ (*x* = 0.01–0.05) nanohexaferrites (NHFs) were synthesized with both sol-gel and ultrasonication approaches. A detailed comparative study of the impact of Tm and Sm co-substitutions on the structural, optical, and magnetic features of Sr-Ba hexaferrites is reported.

## 2. Materials and Methods 

All chemicals were obtained from Merck Co. with high purity and used as received. Strontium nitrate (Sr(NO_3_)_2_, 99%), barium nitrate (Ba(NO_3_)_2_, 99%), iron nitrate (Fe(NO_3_)_3_·9H_2_O, 98%), samarium(III) nitrate hexahydrate (Sm(NO_3_)_3_·5H_2_O, 98%), thulium oxide (Tm_2_O_3_, 99.95%), and citric acid (C_6_H_8_O_7_, 98%) were used as a started material for Sr_0.5_Ba_0.5_Tm*_x_*Sm*_x_*Fe_12−2*x*_O_19_ (*x* = 0.00–0.05) NHFs, which were manufactured via both sol-gel combustion and ultrasonication approaches. Initially, Tm_2_O_3_ was thawed in HCl with heating up to 190 °C under stirring to obtain a transparent solution. In order to synthesize the compositions through sol-gel auto-combustion, a specific ratio of salt nitrate and 10 g of citric acid (C_6_H_8_O_7_) with oxide solution were dissolved in deionized water (DI) under continuous stirring for 45 min with heating at 90 °C. With respect to amending the pH of the mixture, a NH_3_ solution was utilized to set pH at 7, then the temperature was raised first to 180 °C for 40 min and then to 320 °C until the gel was obtained. When the heating process was complete, the gel was burned completely to form a black powder and then calcinated at 1000 °C for 6 h to obtain the pure phase of M-type nanohexaferrite. For the ultrasonic method, the pH of the mixture solutions of metal nitrates and oxides was adjusted to 11 using a NaOH solution. Then, the solution was exposed to ultrasonic irradiation with 20 kHz and 70 W using an ultrasonic homogenizer UZ SONOPULS HD 2070 for 30 min. The final product was rinsed several times with DI water and dried out overnight at 90 °C, then calcinated at 1000 °C for 5 h.

The phase formation was examined by XRD (Rigaku Benchtop Miniflex, Tokyo, Japan) operated with a Cu-Kα line. The morphology, microstructure, EDX, and elemental mapping were carried out via SEM (FEI Titan ST, Hillsboro, OR, USA) and TEM (FEI Titan ST, Hillsboro, OR, USA). FT-IR analyses were done using an ATR Bruker α-II FT-IR spectrophotometer (Bruker, Berlin, Germany). The %DRS measurements were done using a JASCO V-700 UV-Vis spectrophotometer (Shimadzu, Tokyo, Japan). Quantum design PPMS DynaCool-9 (Quantum Design, San Diego, CA, USA) coupled with a vibrating sample magnetometer (VSM) was used for magnetic measurements. 

## 3. Results and Discussion

### 3.1. Phase Identification

**Figure 1:**
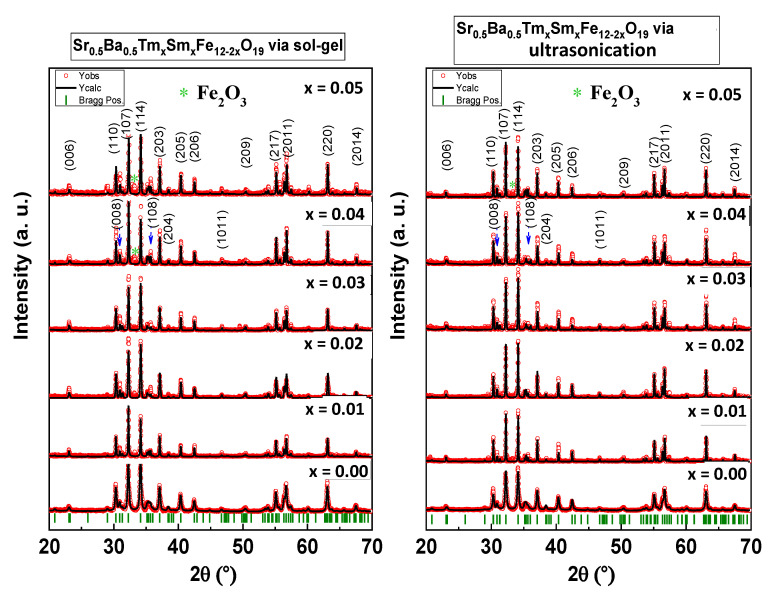
XRD powder patterns of Sr_0.5_Ba_0.5_Tm*_x_*Sm*_x_*Fe_12−2*x*_O_19_ (*x* = 0.00–0.05) nanohexaferrites (NHFs) synthesized via sol-gel combustion and ultrasonication approaches.

Figure 1 shows the XRD patterns of Sr_0.5_Ba_0.5_Tm*_x_*Sm*_x_*Fe_12−2*x*_O_19_ (*x* = 0.00–0.05) NHFs prepared using both sol-gel auto-combustion and ultrasonication techniques. XRD powder patterns of both sol-gel combustion and ultrasonically synthesized products exhibited the indexed peaks of M-type Ba and Sr hexaferrite (ICDD card number 84–0757). It is clear that there is a minor secondary phase of Fe_2_O_3_ (indicated by an asterisk * in Figure 1) at *x* = 0.04 and *x* = 0.05 due to the insertion of different ions in the crystal structure of the hexaferrites. This indicates that the substituted ions are well incorporated into the M-type hexaferrite lattice. The crystallite size and structural parameters (a and c) were calculated by Match 3! and are listed in Table 1. The Rietveld refinement procedure was performed using the FULL PROOF program with multi-phase capability and pseudo-Voight peak shape functions. The *R_Bragg_* factor and the goodness of fit *χ*^2^ were used as the numerical criteria of fitting. For verifying the M-type hexagonal phase of both compositions, c/a ratio was estimated and found to be in the probable range of 3.911–3.924, which is smaller than 3.97 and proves the growth of hexaferrite [21]. The lattice constant “*a*” was found to be almost the same for all ratios, whereas “*c*” increased as the ratio of substitution ions increased, owing to the distortion created by the enlargement of the crystal resulting from the variance in ionic radii of substituted ions in both compositions. The crystal size was evaluated using the Debye–Scherrer equation, taking into consideration the intense peaks of Sr hexaferrite (107) and (114). The crystal sizes were in the range from 26 to 45 nm for both compositions.

### 3.2. Morphological Analysis

**Figure 2:**
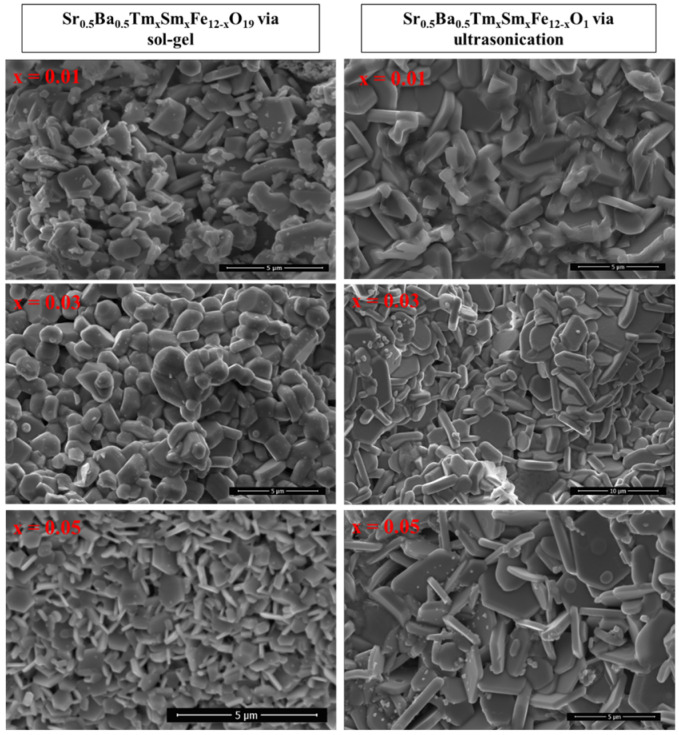
SEM images of Sr_0.5_Ba_0.5_Tm_x_Sm_x_Fe_12−2x_O_19_ (x = 0.01, 0.03, and 0.05) hexaferrites prepared using sol–gel auto-combustion (**left**) and ultrasonication (**right**) techniques.

**Figure 3:**
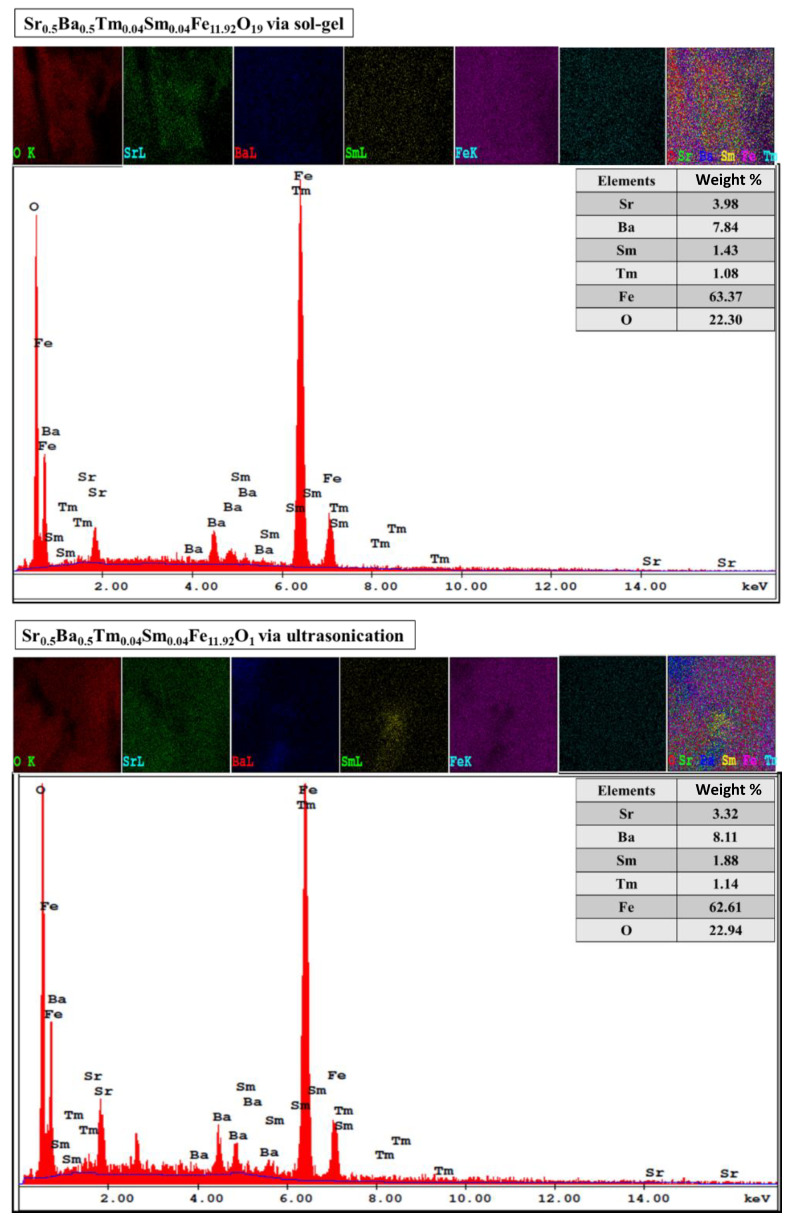
Elemental mappings of Sr_0.5_Ba_0.5_Tm*_x_*Sm*_x_*Fe_12−2*x*_O_19_ (*x* = 0.04) NHFs prepared using both sol-gel auto-combustion and ultrasonication approaches.

**Figure 4:**
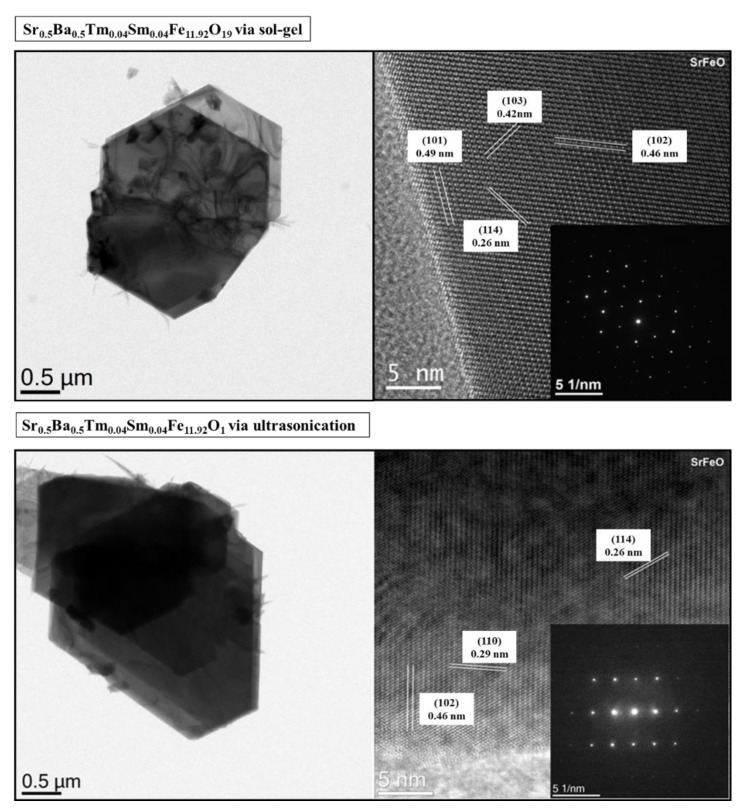
TEM and HR-TEM images of Sr_0.5_Ba_0.5_Tm*_x_*Sm*_x_*Fe_12−2*x*_O_19_ (*x* = 0.04) NHF prepared through both sol-gel auto-combustion and ultrasonication method.

Figure 2 shows the SEM images of Sr_0.5_Ba_0.5_Tm*_x_*Sm*_x_*Fe_12−2*x*_O_19_ (*x* = 0.01, 0.03, and 0.05) NHFs prepared using both sol-gel combustion and ultrasonic techniques. The images reveal the aggregates and randomly oriented platelet-shaped single particles of a few tens of nanometers with obvious boundaries. Moreover, there is a minor difference in the grain size obtained by increasing the substitution level. Figure 3 shows the EDX and elemental mapping of Sr_0.5_Ba_0.5_Tm*_x_*Sm*_x_*Fe_12−2*x*_O_19_ (*x* = 0.04) NHFs prepared through both sol-gel combustion and ultrasonic routes. It can be proven that the elements of both compositions, such as Sr, Ba, Tm, Sm, Fe, and O, nearly reached the original stoichiometric ratios of metal that were used as initial materials. The low-magnification TEM images of Sr_0.5_Ba_0.5_Tm_x_Sm_x_Fe_12−2x_O_19_ (x = 0.04) NHF synthesized using both sol-gel auto-combustion and ultrasonic methods show the typical hexagonal particles., as seen in Figure 4. The HR-TEM observations illustrate the idealistic crystallinity of a typical hexagonal structure with lattice planes (114), (110), (103), (102), and (101) at 0.26, 0.29, 0.42, 0.46, and 0.49, as is clearly seen in Figure 4.

### 3.3. Magnetic Investigations

**Figure 5:**
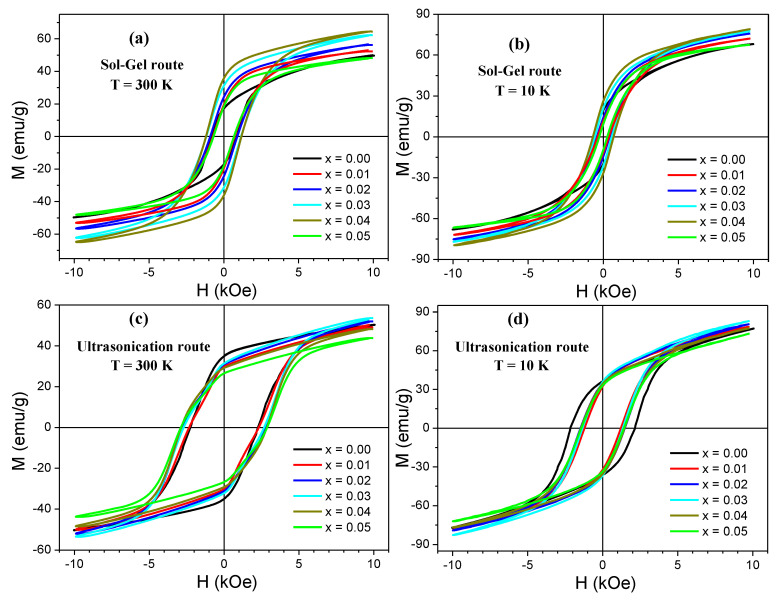
*M-H* hysteresis loops performed at RT and 10 K for different Sr_0.5_Ba_0.5_Tm*_x_*Sm*_x_*Fe_12−2*x*_O_19_ NHFs prepared through (**a**,**b**) sol-gel and (**c**,**d**) ultrasonication routes.

Figure 5 shows the *M*–*H* hysteresis loops performed at RT and 10 K for different Sr_0.5_Ba_0.5_Tm*_x_*Sm*_x_*Fe_12−2*x*_O_19_ (0.00 ≤ *x* ≤ 0.05) NHFs prepared via sol-gel and ultrasonication routes. *M*–*H* curves were carried out up to ±10 kOe external magnetic field. The obtained findings indicated ferrimagnetic (FM) behaviors of various prepared Sr_0.5_Ba_0.5_Tm*_x_*Sm*_x_*Fe_12−2*x*_O_19_ NHFs at both measurement temperatures. As shown in Figure 5, it is evident that the magnetization of each product increased while reducing the temperature from 300 to 10 K. This is principally ascribed to the decline of thermal fluctuations [22,23,24]. This means that a more quantitative ordering of spins at low temperatures and in high magnetic fields contributes to enhancing the magnetic moments. On the other hand, the coercivity diminished while reducing the temperature, which is predominantly recognized by the rise in M_s_ value and the reduction in the effects of magneto-crystalline anisotropy energy with the temperature reduction [22,23]. The magnetization values at maximum applied field of 10 kOe (*M_max_*) for Sr_0.5_Ba_0.5_Tm*_x_*Sm*_x_*Fe_12−2*x*_O_19_ NHFs prepared via sol-gel approach are in the ranges of 48.1–64.6 and 67.5–79.1 emu/g at 300 and 10 K, respectively (Table 2). For samples synthesized through the ultrasonication route, *M_max_* are in the ranges of 43.84–53.36 and 73.30–83.03 emu/g at 300 and 10 K, respectively (Table 2). Nevertheless, it is obvious that an applied field of ±10 kOe is not enough to saturate the prepared samples. Accordingly, Stoner–Wohlfarth (S–W) approximation was utilized to extract *M_s_* values using the following expression [22,23,24]:

(1) $$M=M_{s}\left(1-\frac{b}{H^{2}}\right)$$

**Figure 6:**
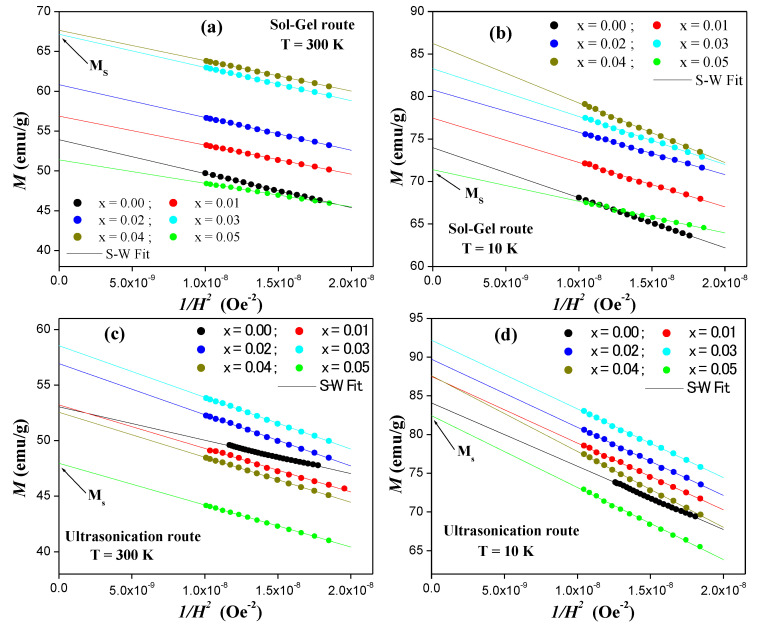
*M* vs. *1/H*^2^ plots performed at RT and 10 K for different Sr_0.5_Ba_0.5_Tm*_x_*Sm*_x_*Fe_12−2*x*_O_19_ NHFs prepared using (**a**,**b**) sol-gel approach and (**c**,**d**) ultrasonication route.

Therefore, by plotting *M* as a function of *1/H*^2^, the value of *M_s_* can be extracted as the value when *1/H*^2^ approaches zero (i.e., the intercept). The slope gives the value of the constant *M_s_b*. *M* versus *1/H*^2^ plots for various Sr_0.5_Ba_0.5_Tm*_x_*Sm*_x_*Fe_12−2*x*_O_19_ NHFs prepared via sol-gel and ultrasonication routes are indicated in Figure 6. The deduced values of the constant *b* are listed in Table 2. The obtained results show that both the substitution of Fe ions by Tm-Sm ions and the synthesis procedures affect significantly the magnetic properties.

**Figure 7:**
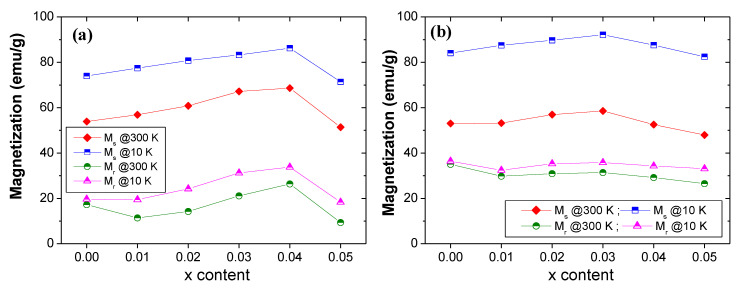
Variations in *M_s_* and *M_r_* with respect to Tm-Sm content at RT and 10 K for various Sr_0.5_Ba_0.5_Tm*_x_*Sm*_x_*Fe_12−2*x*_O_19_ NHFs prepared via (**a**) sol-gel and (**b**) ultrasonication routes.

The changes in *M_s_* and *M_r_* with respect to Tm and Sm contents (*x*) for the different Sr_0.5_Ba_0.5_Tm_x_Sm_x_Fe_12−2x_O_19_ NHFs prepared via sol-gel and ultrasonication routes are presented in Figure 7a,b, respectively. The *M_s_* value of non-substituted SrBa hexaferrite (*x* = 0.00) prepared using the sol-gel technique is equal to 56.87 emu/g at RT. This value is higher than that of the non-substituted sample prepared via ultrasonication route, where the *M_s_* value is around 53.03 emu/g at *T* = 300 K. This is mainly attributed to the difference in crystallite size. Indeed, the ultrasonication route yields a crystallite size smaller than that obtained via sol-gel approach. However, the *M_s_* value is higher at low temperature (*T* = 10 K) for the sample prepared via ultrasonication route compared to that synthesized through sol-gel. This could be ascribed to a more quantitative ordering of spins at low temperatures in samples prepared via ultrasonication route. The same cases were observed for each Tm-Sm substitution. For both samples produced via sol-gel and ultrasonication routes, the *M_s_* values increased with increasing Tm-Sm substitution contents reaching maximum values at a certain level, and then started to decrease with further increasing Tm-Sm contents. The maximum *M_s_* values of 67.65 and 86.24 emu/g at RT and 10 K, respectively, were observed for *x* = 0.04 in the case of samples prepared using the sol-gel technique. However, for those produced via ultrasonication route, the maximum *M_s_* values at both 300 K (*M_s_* = 58.55 emu/g) and 10 K (*M_s_* = 92.18 emu/g) were found for *x* = 0.03 level. The lowest *M_s_* values belonged to the *x* = 0.05 level for both hexaferrites produced using the sol-gel (*M_s_* = 51.38 and 71.38 emu/g at RT and 10 K, respectively) and ultrasonication (*M_s_* = 47.95 and 82.43 emu/g at RT and 10 K, respectively) methods. Nevertheless, the registered magnetization values were greater than those revealed in Co-W-doped SrBa hexaferrites synthesized via ceramic process [25], in SrBa hexaferrites produced using the chemical co-precipitation procedure [26], in Gd-Sn co-substituted HFs [27], and in Co-doped Sr-Ba hexaferrites [28]. The *M_r_* values illustrate practically the same trend as that of the *M_s_* values. In several earlier reports, it was claimed that the trend in *M_r_* depends principally on the tendency of *M_s_* and on the net alignments of grain magnetization caused by strong exchange interactions among nanoparticles (NPs) [29].

M-type hexaferrites comprise five different crystallographic sites of Fe^3+^ ions—one bipyramidal site “2b” having spin-up direction, one tetrahedral site “4f_1_” having spin-down direction, and three octahedral sites amongst which “4f_2_” has spin-down direction and ”12k” and “2a” have spin-up direction [30,31]. The magnetic moments of Fe^3+^ ions are arranged collinearly because of the presence of super-exchange interactions. Generally, numerous factors can influence the magnetic properties of SrBa HFs. The magnetization (M_s_ and *M_r_*) is largely governed by magnetic moments, bulk density, occupation site of doping ions (i.e., compositions), and presence of secondary phases [32,33]. The most reasonable cause for the increase in the *M_s_* values are the magnetic moments of the co-substitutions of Tm^3+^ ions (7.5 μ_B_) and Sm^3+^ ions (1.7 μ_B_). Tm^3+^ ions (7.5 μ_B_) have a magnetic moment larger than that of Fe^3+^ ions (5 μ_B_), which strengthen the super-exchange interactions and hence induce the increase in magnetization while increasing the substitution contents. Moreover, it has been reported that RE elements have the preference of being located in the octahedral sites (2a, 12k, 4f_2_) because of volume effects [34]. Almessiere et al. [23] asserted that a lower substituting content of Tm^3+^ ions has a preference of residing in the 4f_2_ site. In another study, a smaller doping amount of Sm^3+^ ions preferentially occupied the 4f_2_ site [35]. The Fe^3+^ ions at 4f_2_ sites with spin-down direction and those at 12k and 2a sites with spin-up direction have been substituted by Sm^3+^ ions and Tm^3+^ ions, respectively, which results in an increase in the global magnetic moment, and hence the global magnetization of SrBa HFs. Further investigations such as Mossbauer spectroscopy are required in this field, which will be the subject of an upcoming study. Additionally, the evolutions in grain size with substitutions may clarify the variations in the magnetization. The rising tendency of *M_s_* and *M_r_* could be ascribed to the increase of grain size and vice versa. Moreover, Tm-Sm substituting ions improved the density of the produced samples up to *x* = 0.04 for the sol-gel approach and *x* = 0.03 for the ultrasonication route, as shown in the SEM study. This leads to improving the magnetic dipoles per unit volume; hence *M_s_* and *M_r_* rise with substitutions [30]. 

The decrease in magnetization at a high level of substitution is simply attributed to the excess amount of Sm^3+^ ions (1.7 μ_B_) having a lower magnetic moment that that of Fe^3+^ ions (5 μ_B_). This results in a reduction in the total magnetic moment, which weakens the super-exchange interactions and thus decreases the magnetization. At high substituting level, Tm^3+^ and Sm^3+^ ions can also reside in the 2a, 2b, and 12k sites (particularly the 12k site) having upward spins. The total number of unpaired electrons is reduced, resulting in a drop in the values of magnetization. Furthermore, it has been reported that the larger content of rare earth ions gradually alters the magnetization from collinear spins to non-collinear spins of Fe^3+^ ions, and hence decreases the magnetization [30,36]. Sadiq et al. [36] claimed that the doping with RE elements could reduce the strength of super-exchange interactions among Fe^3+^-O-Fe^3+^, leading to spin-canting or non-collinear arrangement of magnetic moments. The decrease in magnetization could be also attributed to the formation of secondary phases of Fe_2_O_3_, as observed in the XRD investigation. The variations in magnetization could be justified based on the dissimilarity in ionic radii of Fe^3+^ (0.645 Å), Tm^3+^ (0.88 Å), and Sm^3+^ (0.958 Å) ions that generates local strains in the hexaferrite system, which in turn produces disorders and variations in the local electronic states [37,38]. 

The experimental values of the magnetic moments *n_B_* can be determined as follows [39]:

(2) $$n_{B}=\frac{Molecular\;Weight\;M_{s}}{5585}$$

*n_B_* values at both RT and 10 K of various hexaferrites prepared via sol-gel and ultrasonication routes are listed in Table 2, respectively. At RT and 10 K, *n_B_* values increased with increasing Tm-Sm substitutions for both samples produced via sol-gel and ultrasonication routes. These values reached their maximums at *x* = 0.04 in the case of samples prepared using the sol-gel technique and at *x* = 0.03 in those produced via ultrasonication route. Afterward, *n_B_* values decreased. The increase/decrease in *n_B_* resulted from the strengthening/weakening of the super-exchange interactions [39]. The observed trend in *n_B_* values is in accordance with the variations in magnetization.

**Figure 8:**
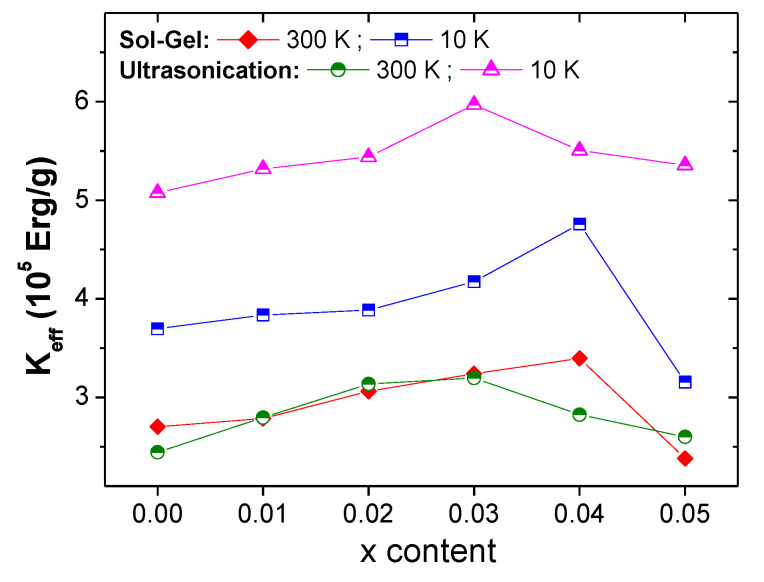
Variations in *K_eff_* with respect to Tm-Sm content at RT and 10 K for different Sr_0.5_Ba_0.5_Tm*_x_*Sm*_x_*Fe_12−2*x*_O_19_ NHFs produced using sol-gel and ultrasonication techniques.

The squareness ratios (*SQR = M_r_/M_s_*) were determined and are listed in Table 2. *SQR* values are below 0.5 at both RT and 10 K for all products prepared using the sol-gel approach, indicating that these materials display multi-magnetic domain regions [40,41]. On the other hand, samples produced via ultrasonication route show *SQR* values above 0.5 at RT for all compositions, revealing that these products exist in the single domain regions at RT [40,41]. However, *SQR* values at 10 K are below 0.5, and these products show multi-magnetic domain regions at low temperature. The effective anisotropy constant (*K_eff_*) with respect to Tm-Sm and elaboration techniques (Figure 8) are deduced as follows [37]:

(3) $$K_{eff}=M_{s}{\left(\frac{15\;b}{4}\right)}^{0.5}$$

The *K_eff_* value shows a similar trend in the evolution of *M_s_.* For samples prepared using the sol-gel approach, *K_eff_* increases with increasing Tm-Sm content up to *x* = 0.04 and then decreases. Similarly, *K_eff_* reaches its maximum for *x* = 0.03 in the case of those prepared via ultrasonication route and then decreases.

**Figure 9:**
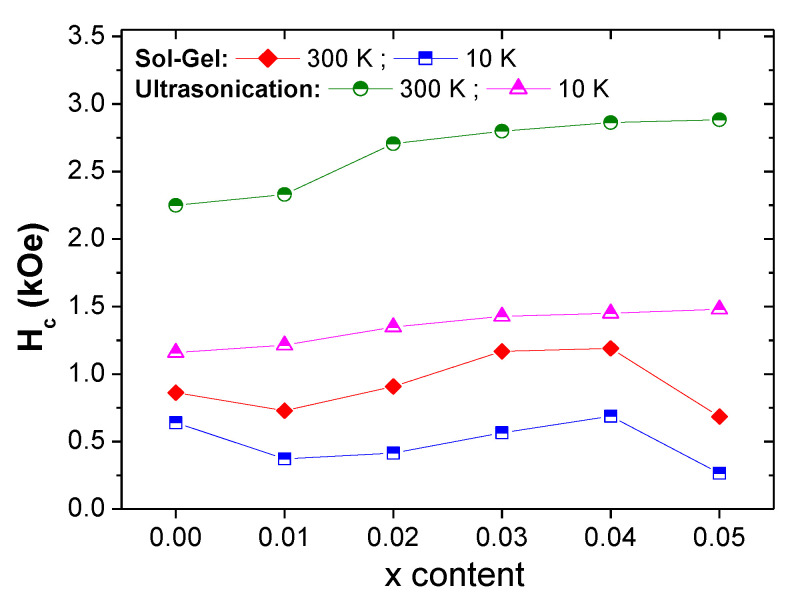
Variations in *H_c_* with respect to Tm-Sm content at RT and 10 K for various samples prepared using sol-gel and ultrasonication routes.

The *M-H* loops for samples prepared via ultrasonication route are very broad in comparison to those produced by means of the sol-gel approach, implying that the products synthesized via ultrasonication route have greater values of coercivity. This could be explained by the fact that the ultrasonication technique leads to the formation of particle sizes smaller than those produced via sol-gel approach. Indeed, the coercivity and the particle size are inversely proportional [39]. The variations in *H_c_* values with respect to Tm-Sm content are shown in Figure 9. For samples produced via ultrasonication route, it can be seen that the coercivity increases continuously with increasing Tm-Sm content. However, in the case of the sol-gel approach, *H_c_* increases up to *x* = 0.04 and then decreases. As mentioned, *H_c_* is correlated to the inverse of particle size and also depends on the magneto-crystalline anisotropy (*K_eff_*) as follows [39]:

(4) $$H_{c}\propto \frac{2K_{eff}}{M_{s}}$$

It has been claimed that by reducing the particle size, *H_c_* is enhanced. However, no clear relation between *H_c_* and *D_XRD_* was found with respect to Tm-Sm substitutions, which is the opposite to what is expected. Therefore, another principal parameter, namely the anisotropy constant, has a major influence on the variations in *H_c_* values with respect to Tm-Sm substitutions. As shown in Figure 8 and Figure 9, the observed increase in the *H_c_* values is due to the increase in magneto-crystalline anisotropy (*K_eff_*). As shown in the XRD study, hexaferrite samples having a higher Tm-Sm substituting level contain secondary phases of Fe_2_O_3_, which causes a reduction in the magneto-crystalline anisotropy. Consequently, this leads to a decrease in *H_c_* values [42].

### 3.4. Optical Properties

**Figure 10:**
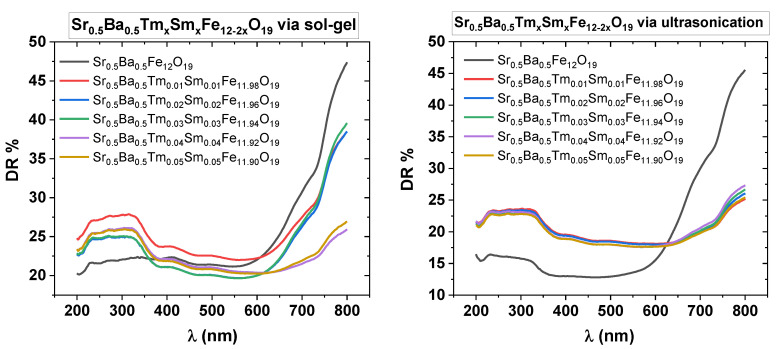
DR % vs. λ spectra of Sr_0.5_Ba_0.5_Tm*_x_*Sm*_x_*Fe_12−2*x*_O_19_ (*x* = 0.00–0.05) NHFs prepared via sol-gel and ultrasonication techniques in the range of 200–800 nm.

Diffuse reflectance spectra (DR %) recorded for Sr_0.5_Ba_0.5_Tm*_x_*Sm*_x_*Fe_12__−2*x*_O_19_ (*x* = 0.00–0.05) NHFs prepared via both sol-gel auto-combustion and ultrasonication techniques are shown in Figure 10. Spectra were recorded using a UV-Vis spectrophotometer equipped with an integrating sphere to pick up the diffused and scattered reflections from powder samples. From the figure, one can easily conclude that the DR magnitude of co-doped ensembles are up to 10% larger with respect to DR of undoped Sr_0.5_Ba_0.5_Fe_12_O_19_ samples in the photometer’s sweep range between 200 nm and 600 nm. On the contrary, the reflectance magnitudes of undoped Sr_0.5_Ba_0.5_Fe_12_O_19_ samples are remarkably higher than the reflectance of co-doped NHFs above 650 nm.

**Figure 11:**
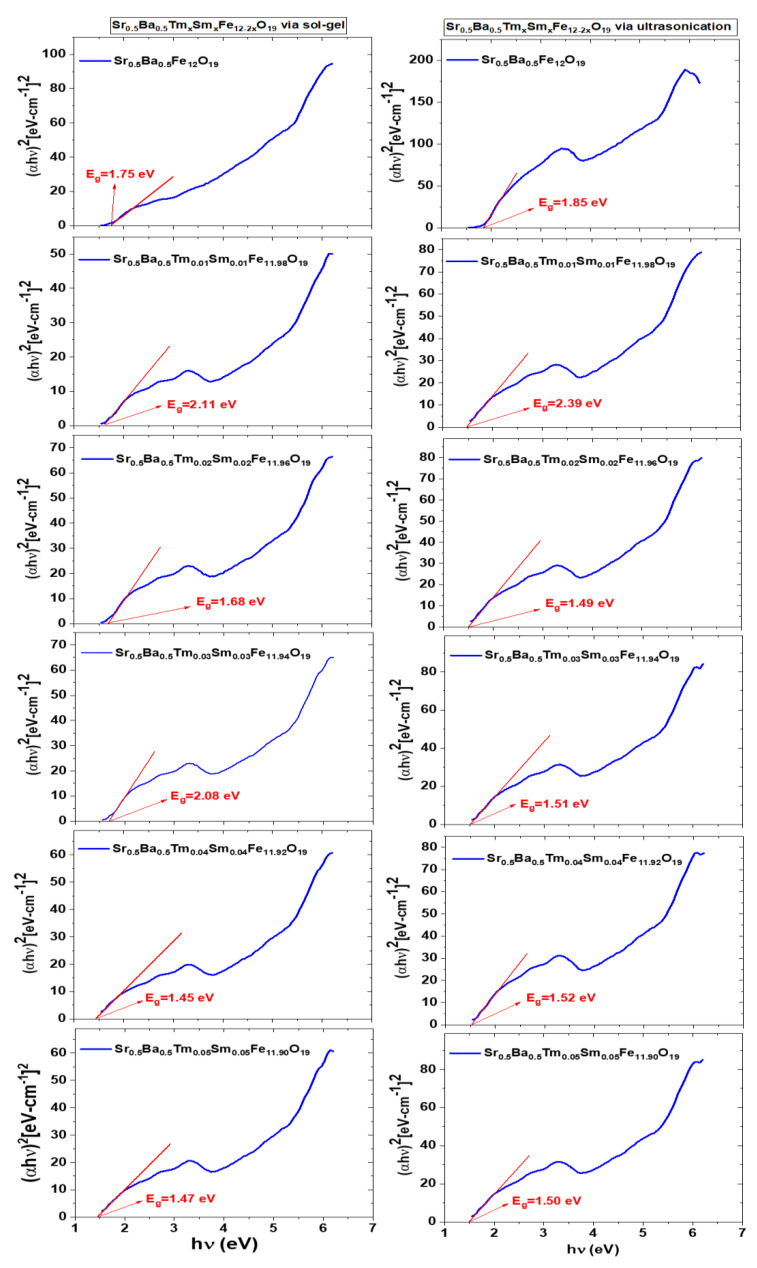
Tauc plots and extrapolated *E*_g_ data of Sr_0.5_Ba_0.5_Tm*_x_*Sm*_x_*Fe_12__−2*x*_O_19_ (*x* = 0.00–0.05) NHFs prepared via sol-gel and ultrasonication techniques.

Initial scientific background for optical energy band gap (*E*_g_) determination from DRS data for powder nanoparticle samples was formed using the Kubelka–Munk theory [43,44,45]. *E*_g_ values are estimated applying the expression called the Tauc equation as follows:

(5) $${\left(\alpha h\upsilon \right)}^{\frac{1}{n}}=A\left(h\upsilon -E_{g}\right)$$

where α is the absorption coefficient, *hυ* is the energy of the incident beam of light, *A* is a proportionality constant, and power *n* is equal to 1/2 denoting the direct electronic transitions, respectively. (*αhυ*)^2^ *vs hυ* plots are drawn and linear parts of the plots that are close to the energy axis are extrapolated until the intersecting energy axis. Intercepts correspond to the direct band gap magnitude of samples in units of eV, as indicated in Figure 11. Plots that are in the left column of the figure belong to the samples fabricated by the sol-gel approach and the plots in the right column belong to samples fabricated by the ultrasonication approach. Both undoped Sr_0.5_Ba_0.5_Fe_12_O_19_ samples that were synthesized by sol-gel and ultrasonication approaches have the maximum *E*_g_ values of 1.75 eV and 1.85 eV, respectively. The co-doping process significantly decreases those maxima. *E*_g_ of the Sr_0.5_Ba_0.5_Tm_0.04_ Sm_0.04_Fe_11.94_O_19_ sample, which was synthesized by the sol-gel method, has *E*_g,min_ = 1.45 eV in its group. On the other hand, samples Sr_0.5_Ba_0.5_Tm_0.01_ Sm_0.01_Fe_11.98_O_19_ and Sr_0.5_Ba_0.5_Tm_0.02_ Sm_0.02_Fe_11.96_O_19_, which were synthesized by the ultrasonication method, have the same *E*_g,min_ values of 1.49 eV in their group. There is almost a steady dropping rate of band gap values for co-doped samples synthesized by the sol-gel method. However, we observed a sharp drop to an average of 1.50 eV for the co-doped samples synthesized by the ultrasonication method. Sudden observed drops of the *E*_g_ values for Sr_0.5_Ba_0.5_Tm*_x_*Sm*_x_*Fe_12−2*x*_O_19_ NHFs that include *x*= 0.01 and 0.02 co-doped ion contents may be ascribed to the creation of sub-bands in the forbidden gap and the subsequent assimilation with the conduction bands to perform as a continuous band structure [46,47]. A further decrease with increasing co-doped ion content especially observed for the products that were fabricated by the sol-gel method is an expected case. The increasing carrier concentration narrows the band gap between the top level of the valence band and the minimum level of the conduction band. As a group, we reported a large quantity of *E*_g_ data in the range of 1.74–2.0 eV for SrFe_12_O_19_ and BaFe_12_O_19_ hexaferrites. Single ion-doped or co-doped samples with many ions such as Cu^2+^, Mn^2+^, La^2+^, Zr^2+^, Bi^3+^, La^3+^, Y^3+,^ Cr^3+^, Tb^3+^, and Tm^3+^ have an *E*_g_ range between 1.34 eV and 2.15 eV [22,40,48,49,50]. Different synthesis methods were applied such as hydrothermal route, sonochemical approach, or sol-gel auto-combustion. However, we did not find in the literature any reported *E*_g_ data about Sr_0.5_Ba_0.5_Fe_12_O_19_ NHFs especially co-doped with Tm^3+^ and Sm^3+^ ions.

## 4. Conclusions

Sol-gel auto-combustion and ultrasonic methods were employed to synthesize the Sr_0.5_Ba_0.5_Tm*_x_*Sm*_x_*Fe_12−2*x*_O_19_ (*x* = 0.00–0.05) samples. XRD revealed the creation of an M-type hexaferrite structure for both compositions with crystal size in the range of 26 to 45 nm. SEM, TEM, and HR-TEM confirmed the hexagonal platelet morphology. *M-H* loops indicated the ferrimagnetic nature of various prepared hexaferrites via sol-gel and ultrasonication routes. The *M_s_* and *M_r_* values increased with increasing Tm-Sm substituting contents. *M_s_* and *M_r_* reached their maximum values at *x* = 0.04 in the case of samples prepared using the sol-gel technique and at *x* = 0.03 for those prepared via ultrasonication route. The magnetization was found to be largely governed by magnetic moments, bulk density, occupation site of doping ions (i.e., compositions), and presence of secondary phases. *M-H* loops were very broad in samples prepared via ultrasonication route in comparison to those produced by means of the sol-gel approach, implying that the products synthesized via ultrasonication route have greater values of coercivity (*H_c_*). *H_c_* increased continuously with increasing Tm-Sm content for samples produced via ultrasonication route. However, in the case of the sol-gel approach, *H_c_* increased up to *x* = 0.04 and then decreased. The variations in *H_c_* values with respect to Tm-Sm substitutions were governed by the evolutions in the magneto-crystalline anisotropy. Direct *E*_g_ data of pristine Sr_0.5_Ba_0.5_Fe_12_O_19_ ensembles that were synthesized by both methods are significantly tuned by the co-substitution of Tm^3+^ and Sm^3+^ ions. Estimated band gap energies between minimum 1.45 eV and maximum 1.85 eV assign our samples as semiconducting materials.

## Figures and Tables

**Table 1:** The refined structural parameters of Sr_0.5_Ba_0.5_Tm_x_Sm_x_Fe_12−2x_O_19_ prepared via sol-gel combustion and ultrasonication approaches.

Hexaferrites Prepared via Sol-Gel Approach							
*x*	*a = b* (Å)	*c* (Å)	*V* (Å)^3^	*c/a*	*D_XRD_* (±0.05 nm)	*χ*^2^ (*chi*^2^)	*R_Bragg_*
0.00	5.8879(4)	23.1126(3)	693.9162	3.92	36.4	3.14	11.2
0.01	5.8903(5)	23.1252(5)	694.8590	3.92	39.5	2.73	24.3
0.02	5.8920(6)	23.1656(3)	696.9880	3.93	44.7	5.51	26.1
0.03	5.8890(4)	23.1820(6)	697.9178	3.93	45.1	4.00	25.3
0.04	5.8920(6)	23.1830(6)	698.3829	3.93	46.0	3.30	28.1
0.05	5.8900(5)	23.1940(5)	698.9847	3.93	40.6	3.50	22.5
**Hexaferrites Prepared via Ultrasonication Approach**							
***x***	***a = b* (Å)**	***c* (Å)**	***V* (Å)^3^**	***c/a***	***D_XRD_* (±0.05 nm)**	***χ*^2^ (*chi*^2^)**	***R_Bragg_***
0.00	5.8883(5)	23.1249(3)	694.3737	3.92	24.3	3.90	12.9
0.01	5.8906(3)	23.1275(4)	694.9875	3.92	26.9	3.00	25.8
0.02	5.8875(3)	23.1311(6)	694.3753	3.92	31.1	2.40	23.9
0.03	5.8897(6)	23.1332(4)	694.9580	3.92	31.2	3.30	26.0
0.04	5.8917(4)	23.1351(6)	695.4707	3.92	24.1	2.80	26.5
0.05	5.8914(4)	23.1354(4)	695.4277	3.92	23.4	3.60	21.2

**Table 2:** The deduced magnetic parameters at both *T* = 300 and 10 K of various Sr_0.5_Ba_0.5_Tm*_x_*Sm*_x_*Fe_12−2*x*_O_19_ NHFs prepared via sol-gel and ultrasonication routes.

Hexaferrites Prepared via Sol-Gel Approach										
*x* Content	*M_H=10 kOe_ (emu/g)*		*SQR*		*b (10^6^ Oe* ^2^ *)*		*n_B_ (* *μ_B_)*		*H_a_ (kOe)*	
	300 K	10 K	300 K	10 K	300 K	10 K	300 K	10 K	300 K	10 K
0.00	52.3	72.2	0.320	0.266	6.70	6.66	10.49	14.39	10.0	10.0
0.01	52.3	72.2	0.342	0.147	6.40	6.54	11.09	15.10	9.8	9.9
0.02	56.4	75.7	0.399	0.176	6.76	6.17	11.88	15.77	10.1	9.6
0.03	62.3	77.5	0.465	0.253	6.21	6.77	13.14	16.30	9.6	10.0
0.04	64.6	79.1	0.492	0.306	6.53	8.12	13.46	16.91	9.2	11.0
0.05	48.1	67.5	0.357	0.131	5.72	5.21	10.09	14.02	9.3	8.8
**Hexaferrites Prepared via Ultrasonication Approach**										
***x* Content**	***M_H=10 kOe_ (emu/g)***		***SQR***		***b (10^6^ Oe*** **^2^** ***)***		***n_B_ (*** ***μ_B_)***		***H_a_ (kOe)***	
	**300 K**	**10 K**	**300 K**	**10 K**	**300 K**	**10 K**	**300 K**	**10 K**	**300 K**	**10 K**
0.00	50.33	76.41	0.660	0.433	5.66	9.72	10.32	16.36	9.2	12.1
0.01	50.24	78.56	0.560	0.370	7.36	9.85	10.37	17.06	10.5	12.2
0.02	51.92	80.65	0.543	0.393	8.09	9.80	11.12	17.52	11.0	12.1
0.03	53.36	83.03	0.536	0.388	7.95	11.18	11.46	18.04	10.9	12.9
0.04	48.18	77.48	0.556	0.392	7.71	10.53	10.30	17.17	10.7	12.5
0.05	43.84	73.30	0.553	0.402	7.84	11.26	9.42	16.19	10.8	13.0
