# Correction: Slimani et al. Impacts of Sol–Gel Auto-Combustion and Ultrasonication Approaches on Structural, Magnetic, and Optical Properties of Sm-Tm Co-Substituted Sr_0.5_Ba_0.5_Fe_12_O_19_ Nanohexaferrites: Comparative Study. *Nanomaterials* 2020, *10*, 272

**DOI:** 10.3390/nano16040226

**Published:** 2026-02-10

**Authors:** Yassine Slimani, Munirah Abdullah Almessiere, Sadik Güner, Umran Kurtan, Abdulhadi Baykal

**Affiliations:** 1 Department of Biophysics, Institute for Research and Medical Consultations (IRMC), Imam Abdulrahman Bin Faisal University, P.O. Box 1982, Dammam 31441, Saudi Arabia; 2 Institute of Inorganic Chemistry, RWTH Aachen University, D-52074 Aachen, Germany; 3 Department of Materials and Materials Processing Technologies, Vocational School of Technical Sciences, İstanbul University-Cerrahpaşa, 34500 İstanbul, Turkey; 4 Department of Nanomedicine, Institute for Research and Medical Consultations (IRMC), Imam Abdulrahman Bin Faisal University, P.O. Box 1982, Dammam 31441, Saudi Arabia

In the published publication [1], the authors regret to inform that they noticed an unintentional duplication between the SEM image of x = 0.05 in Figure 2 and x = 0.03 sample in Figure 3 of the publication in Materials Chemistry and Physics [2]. The duplicated SEM image in publication [2] was corrected (10.1016/j.matchemphys.2024.130297).

In the original Nanomaterials publication, no change in the SEM image is required; however, a mistake in the published scale bars should be corrected. The corrected Figure with updated scale bars appears below. 

**Figure 2:**
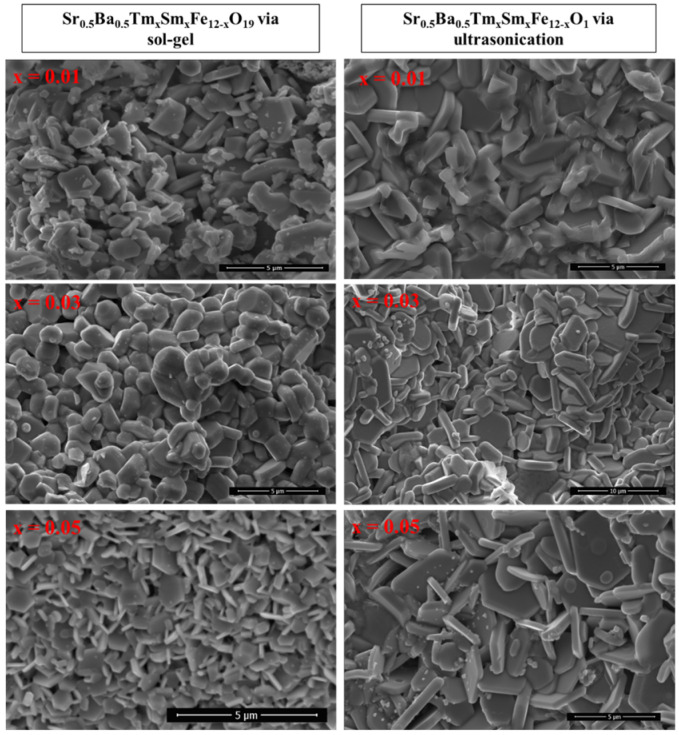
SEM images of Sr_0.5_Ba_0.5_Tm_x_Sm_x_Fe_12−2x_O_19_ (x = 0.01, 0.03, and 0.05) hexaferrites prepared using sol–gel auto-combustion (**left**) and ultrasonication (**right**) techniques.

The slight change in SEM scale bars had no influence on any other results reported in the published Nanomaterials article. The variations in average values of grain size are in good agreement with the original interpretations reported in the “Nanomaterials” article.

In order to avoid any confusion between the two corrections, the authors want to clarify the following points:

- The unintentional duplication occurred due to the very close proximity in composition of the samples investigated in the two papers, and the near simultaneous period of manuscript preparation.
- While the compositions studied in the two papers look very close to each other, they are not exactly identical: Sr_0.5_Ba_0.5_Tm_x_Tb_x_Fe_12−2x_O_19_ (*x* = 0.00–0.05) is in the “Materials Chemistry and Physics” article, and Sr_0.5_Ba_0.5_Tm_x_Sm_x_Fe_12−2x_O_19_ (*x* = 0.00–0.05) is in the “Nanomaterials” article. Although all the dopants are rare-earth elements, they likely influence the physical properties of hexaferrites differently due to their distinct characteristics. Furthermore, the synthesis conditions are slightly different, which will also slightly affect the properties and morphologies of final products. The slight differences in dopants and synthesis conditions (e.g., calcination temperature) could indeed lead to variations in the resulting microstructures and grain sizes. Therefore, a direct comparison of the absolute values of properties, such as average grain size, across the two studies may not be scientifically sound or expected to yield a perfectly consistent trend. Accordingly, a direct comparison between the two studies is not appropriate.
- The purpose of the Corrigendum in the “Materials Chemistry and Physics” article was to rectify the image duplication by providing the correct SEM image for the x = 0.03 composition related exactly to that study. As the original version of “Materials Chemistry and Physics” article involves grain size histogram, and because the SEM image was updated, it was consequently necessary to update its related grain size histogram. The scale bars are correct in the Corrigendum.
- The apparent non-monotonous trend observed when trying to compare data across the Corrigendum “Materials Chemistry and Physics” article could be due to the fact that the sampling area used for generating this histogram in the Corrigendum have been biased towards a region with smaller grains, leading to the lower average grain size. Also, the apparent non-monotonous trend observed when trying to compare data across the “Materials Chemistry and Physics” article likely arises from the fact that we are dealing with a system doped with different concentrations or Tm and Tb rare-earth elements. The subtle variations in the concentration of dopants can significantly influence the resulting microstructure.
- The average grain size derived from the histogram in the Corrigendum of “Materials Chemistry and Physics” article appears smaller than the values in the “Nanomaterials” article. However, this difference should be interpreted within the understanding that these are results from different material compositions and synthesis conditions. Therefore, a direct comparison of average grains size and other properties between the two studies is not appropriate.
- The average grain size histograms for the “Nanomaterials” article are also prepared and presented below (Figure 1). As can be seen, the variations in average values of grain size are in good agreement with the original interpretations reported in the “Nanomaterials” article, which confirms that the slight change in SEM scale bars had fortunately no influence on any other results reported in the published “Nanomaterials” article.

The authors state that the scientific conclusions are unaffected. The authors would like to apologize for any inconvenience caused. This correction was approved by the Academic Editor. The original publication has also been updated.

## Figures and Tables

**Figure 1:**
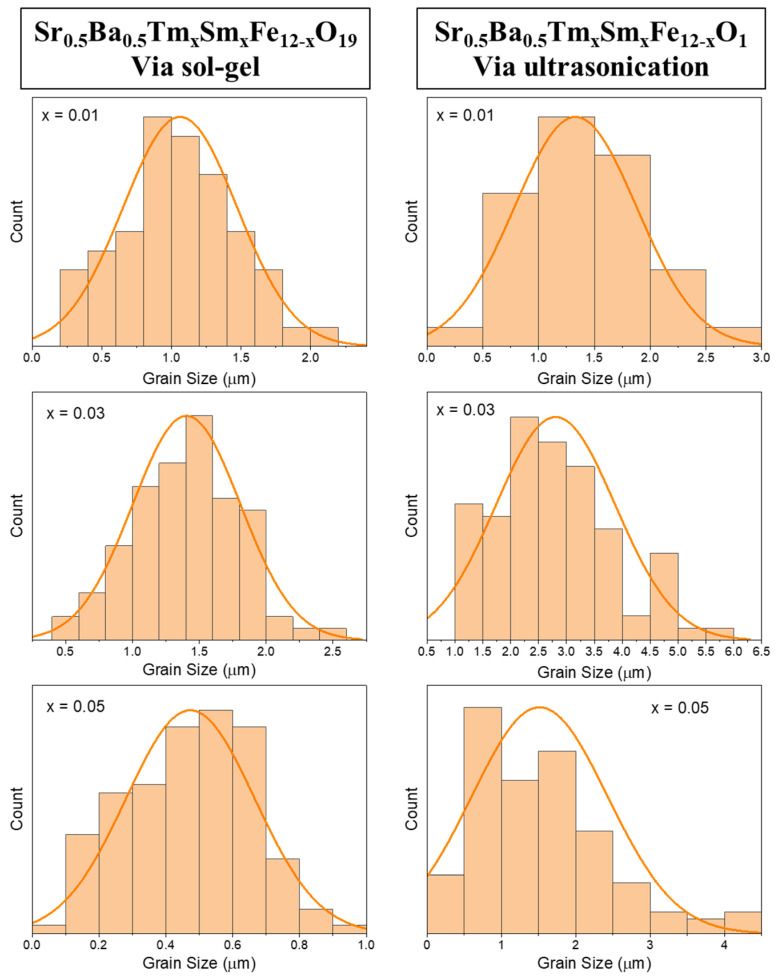
Tm*_x_*Sm*_x_*Fe_12−2*x*_O_19_ (x = 0.01, 0.03, and 0.05) hexaferrites prepared using sol–gel auto-combustion (**left**) and ultrasonication (**right**) techniques.
